# The role and application of small extracellular vesicles in breast cancer

**DOI:** 10.3389/fonc.2022.980404

**Published:** 2022-09-14

**Authors:** Xiaomei Yi, Defa Huang, Zhengzhe Li, Xiaoxing Wang, Tong Yang, Minghong Zhao, Jiyang Wu, Tianyu Zhong

**Affiliations:** ^1^ The First School of Clinical Medicine, Gannan Medical University, Ganzhou, China; ^2^ Laboratory Medicine, First Affiliated Hospital of Gannan Medical University, Ganzhou, China

**Keywords:** small extracellular vesicles, breast cancer, biogenesis, molecular mechanisms, diagnosis, treatment

## Abstract

Breast cancer (BC) is the most common malignancy and the leading cause of cancer-related deaths in women worldwide. Currently, patients’ survival remains a challenge in BC due to the lack of effective targeted therapies and the difficult condition of patients with higher aggressiveness, metastasis and drug resistance. Small extracellular vesicles (sEVs), which are nanoscale vesicles with lipid bilayer envelopes released by various cell types in physiological and pathological conditions, play an important role in biological information transfer between cells. There is growing evidence that BC cell-derived sEVs may contribute to the establishment of a favorable microenvironment that supports cancer cells proliferation, invasion and metastasis. Moreover, sEVs provide a versatile platform not only for the diagnosis but also as a delivery vehicle for drugs. This review provides an overview of current new developments regarding the involvement of sEVs in BC pathogenesis, including tumor proliferation, invasion, metastasis, immune evasion, and drug resistance. In addition, sEVs act as messenger carriers carrying a variety of biomolecules such as proteins, nucleic acids, lipids and metabolites, making them as potential liquid biopsy biomarkers for BC diagnosis and prognosis. We also described the clinical applications of BC derived sEVs associated MiRs in the diagnosis and treatment of BC along with ongoing clinical trials which will assist future scientific endeavors in a more organized direction.

## Introduction

BC is the leading cause of cancer-related deaths worldwide and is the most common and deadliest invasive cancer among women (1). BC is a heterogeneous disease that exhibits extensive genomic, transcriptomic and proteomic alterations (2). Obesity, smoking and lack of physical activity are the most common causes of BC. Conventional screening methods encompassing mammography, tissue biopsy investigations and the therapy approaches including surgery, chemo-, radiation-, and hormone therapy have definitely improved BC survival (3). However, BC still faces a high rate of invasion, metastasis, recurrence and drug resistance (4), which poses a significant burden to families and society. Therefore, exploring new biological mechanisms and new diagnostic and therapeutic methods are still important to reduce the burden of BC.

In recent years, an increasing number of studies have shown that sEVs play an important role in intercellular communication and maintenance of cancer cells homeostasis. sEVs are secreted by almost all cells and are widely present in various body fluids, including urine (5), blood (6), milk (7), saliva (8), cerebrospinal fluid (9), amniotic fluid (10) and semen (11). Exosomes are defined as a subset of sEVs with a diameter of 30-200 nm (12). However, due to EVs heterogeneity, it is difficult to determine their pathways of origin and composition of surface proteins. Therefore, International Society for Extracellular Vesicles (ISEV) published the Minimal information for studies of extracellular vesicles 2018 (MISEV2018) that recommend the use of “small extracellular vesicles (sEVs)” as the current term (13). Since sEVs carry parental information and contain cargo molecules such as nucleic acids, proteins and lipids, these cargo molecules act as mediators of intercellular communication locally and systemically by inducing phenotypic changes in the recipient cells (14). Importantly, they regulate intracellular pathways involved in various stages of BC development, thereby mediating BC cells proliferation, migration, invasion, immune evasion, and drug resistance (15, 16). Furthermore, due to variations in cargo molecules, a series of studies found that sEVs have great potential as non-invasive biomarker carriers for BC diagnosis and prognosis (17, 18). For example, miRNAs wrapped in sEVs, such as miR-1246 and miR-21, are candidate biomarkers for early detection of BC (19, 20). Besides, sEVs have also been explored as nanodrug delivery systems to deliver anticancer drugs to alter cancer cells gene expression (21–23). Here, we reviewed current research, with a particular focus on the role played by sEVs in the development of BC. Additionally, we will discuss the future of clinical applications of sEVs in BC, including their use as diagnostic and prognostic biomarkers and therapeutic targets.

## sEVs biogenesis

**Figure 1:**
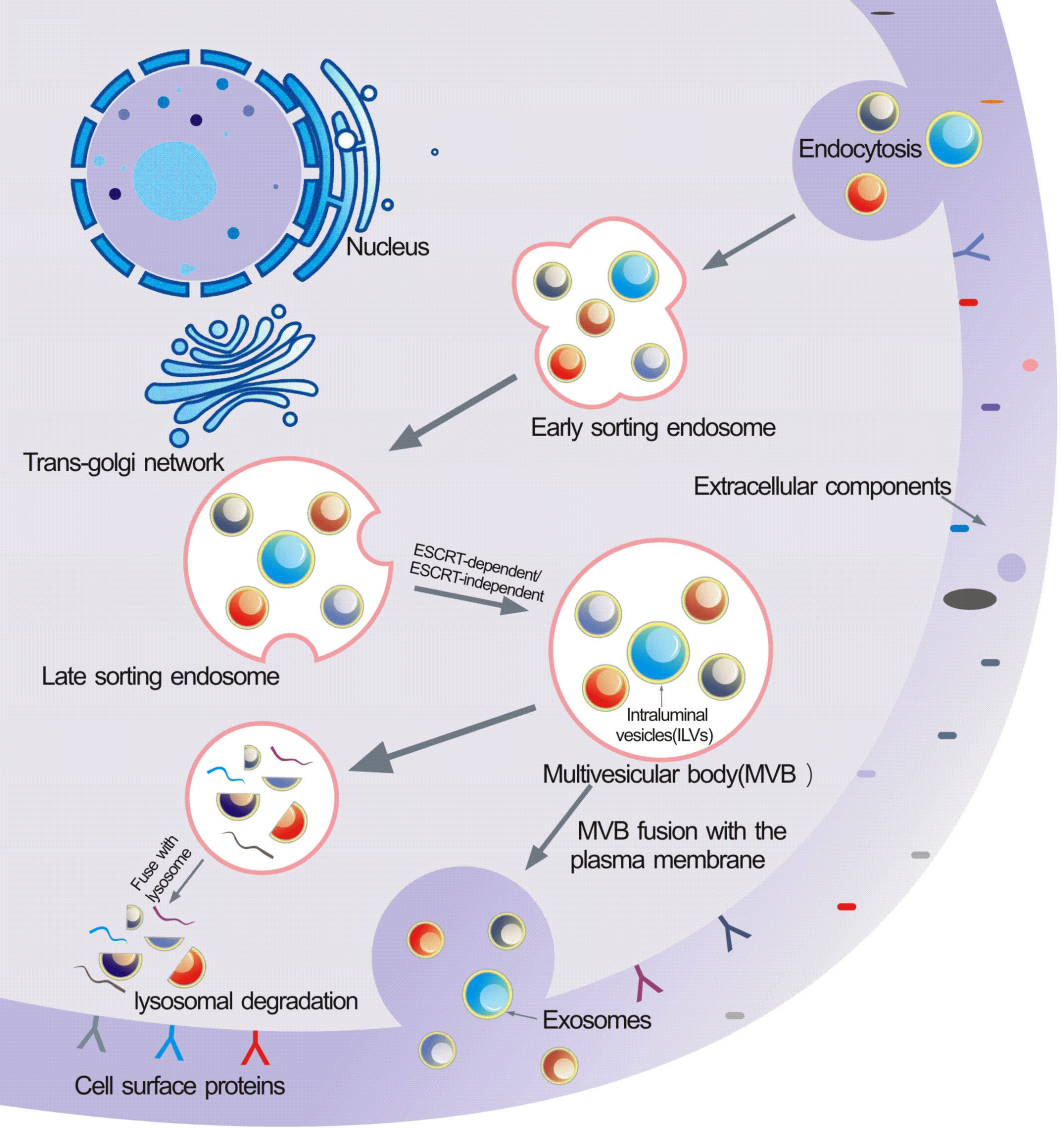
Schematic illustration of exosomes biogenesis. Extracellular constituents along with cell surface proteins enter cells through endocytosis of plasma membrane segments. Early sorting endosome (ESE) are formed after the inward budding of the plasma membrane, then they transport from ESE to late sorting endosome (LSE).Invagination in the LSE results in the multivesicular body(MVB)generation containing Intraluminal vesicles(ILVs).Several machineries including ESCRT-dependent and ESCRT-independent are involved in this process. MVB then can either fuse with lysosomes for degradation or be released into the extracellular space by fusing with the plasma membrane.

**Figure 2:**
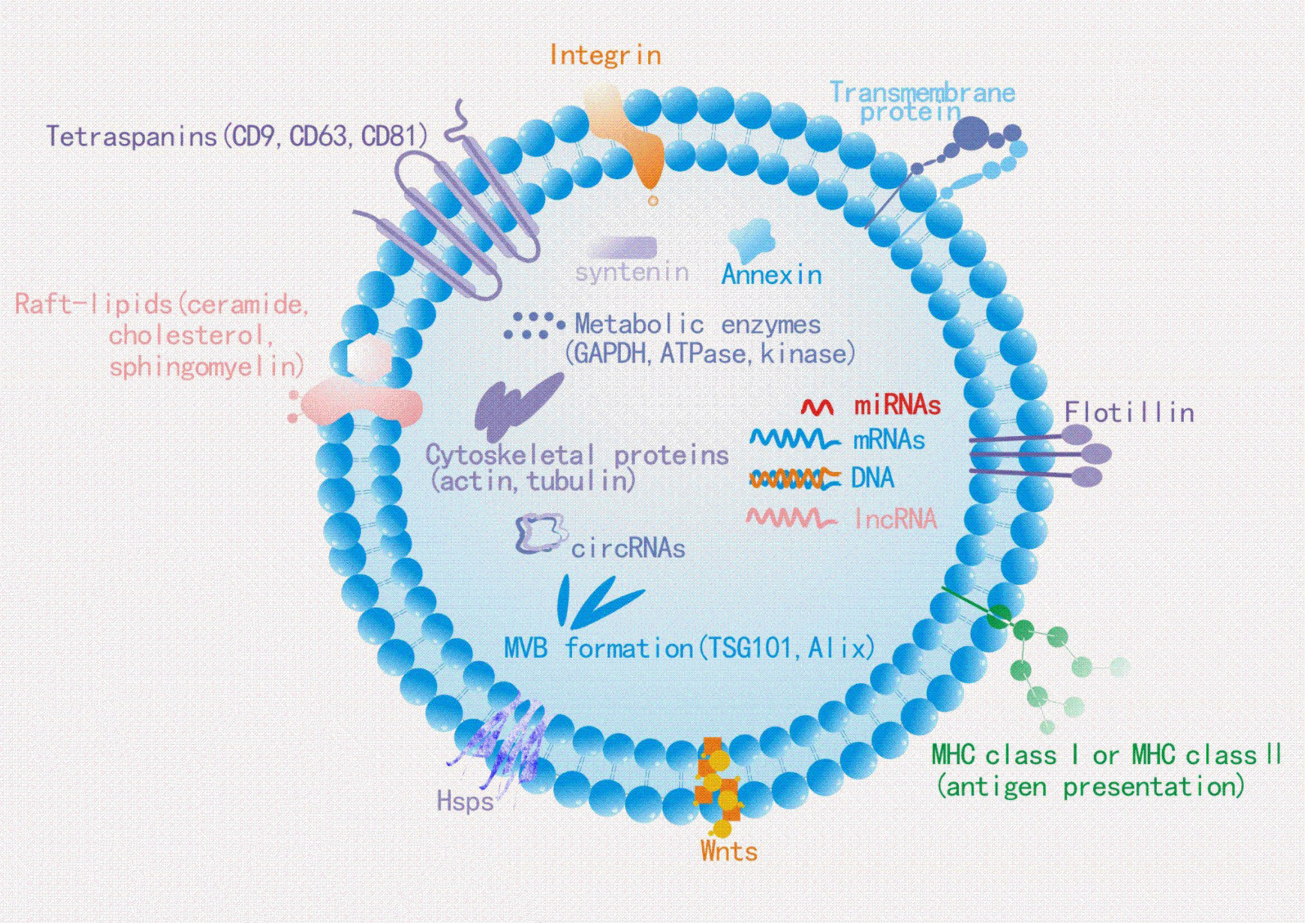
Typical exosome structure: Exosomes are surrounded by a phospholipid bilayer and contain different types of cell surface proteins, lipids, intracellular proteins, DNA, RNA, and metabolites. Several molecules are used as exosome markers (CD9, CD63, CD81, TSG101, and ALIX).

The biogenesis of sEVs is an extremely complex process and involves multiple mechanisms (**Figure 1**). It begins with the endocytosis pathway, which consists of the invagination of the plasma membrane to wrap together cell membrane proteins and some extracellular components to form early sorting endosomes (ESEs) (24, 25). Subsequently, early sorting endosomes exchange materials with other organelles or mature further into late sorting endosomes (LSEs), and late sorting endosomal membranes invaginate to form multivesicular bodies (MVBs) containing intraluminal vesicles (ILVs) (26, 27). After that, MVBs bind to lysosomes or autophagosomes for degradation, or are transported to the plasma membrane through the cytoskeleton and microtubule network, where they fuse with the plasma membrane and exocytose to form sEVs (28–30). Among them, formation of ILVs, avoid degradation of MVBs and fusion of MVBs with the plasma membrane are three crucial processes in the biogenesis of sEVs (31–33). Notably, sEVs cargo molecules(**Figure 2**) are essential elements of the biogenesis mechanisms in these processes, including Ras-related proteins Rab GTPases, Syntenin, ESCRT proteins, HSP proteins, four transmembrane proteins (CD9, CD63, CD81, CD82) and lipids (34–37). For instance, the cytokinesis of ILV-containing MVBs is mainly driven by Rab GTPases at the plasma membrane, particularly RAB27A and RAB27B (38, 39). In particular, lipid molecules in sEVs affect membrane fluidity or curvature through their structural properties and metabolic characteristics, which in turn promote membrane invagination and induce spontaneous outgrowth of ILVs (28, 40, 41).

It is generally believed that the mechanism of sEVs formation process mainly includes two pathways: ESCRT-dependent pathway and ESCRT-independent pathway. The role of the ESCRT complex in the biogenesis of sEVs was recently found to be mainly in promoting the outgrowth and breakage of ILVs and releasing them into the nuclear endosomes (42). ESCRT consists of 4 different protein complexes (ESCRT-0, -I, -II and -III) that bind to VPS4, Alix, and VTA1 proteins to facilitate the formation and transport of MVBs (43). Initially, ESCRT-0 is recruited to the endosomal membrane by monoubiquitinated transmembrane proteins to promote the microdomain aggregation of ESCRT-0 in the vacuolar fraction of the nuclear endosome and to recruit ESCRT-1 through the interaction of the HRS PSAP structural domains with the subunit of ESCRT-1, TSG101 (44). Afterwards, ESCRT-0 and ESCRT-I aggregate cargoes under a flat lattice proteins coating to form the substructural domains of the endosomal membrane (45, 46). When ESCRT-I/II triggers the initial internal rotation of the boundary membrane into the MVBs cavity, ESCRT-III forms a spiral structure that constricts the neck of the sprout (47, 48). Once mature, MVBs can fuse with the plasma membrane to release sEVs into the extracellular space or fuse with lysosomes to degrade their cargoes (49). Notably, sEVs marker protein ALIX has been reported to collaborate with other ESCRT proteins such as TSG101 and CHMP4 to promote cargo sorting, endosomal membrane outgrowth and vesicle detachment in the form of syntenin-syndecan-ALIX complexes (50). However, several studies have shown that lipid raft microdomains play a critical role in sEVs biogenesis independent of ESCRT. Ceramide is a conical lipid whose secretion depends on the action of neutral sphingomyelinase, and once ceramide is generated from sphingolipids, it is readily converted to other bioactive sphingolipids such as sphingomyelin and sphingosine 1-phosphate (51). Trajkovic et al. reported that in oligodendrocytes, inhibition of sphingomyelinase, but not ESCRT depletion, significantly reduced the formation of sEVs, a process known as “ceramide-dependent sEVs biogenesis” (52). Further studies suggest that ceramide is not directly involved in the maturation of MVBs and the formation of sEVs, which may be related to S1P. S1P is a sphingosine phosphorylation product catalyzed by sphingosine kinase (SphK) and an essential component of the formation and maturation of MVBs (53). Interestingly, some tetra-transmembrane proteins such as CD9, CD63, CD81 and CD82 are thought to play a vital role in the formation of sEVs in an ESCRT-independent manner. CD63-dependent mechanisms have been reported to stimulate the production of melanosome-rich sEVs (31). Similarly, elevated expression of CD9 and CD82 increased the release of β-catenin rich in sEVs from human embryonic kidney 293 cells (HEK293) (54). None of the currently known biogenesis mechanisms are exclusively specific to sEVs pathway, nor are any of them present in all cell types. The crucial questions that need to be urgently addressed now are to find cell-specific sEVs and to clarify how their contents interact in the generation of sEVs.

Overall, the regulation of sEVs biogenesis involves the coordination of many different molecular cargoes and signaling mechanisms, dominated by ESCRT-dependent mechanism, lipid rafts and four-transmembrane protein mechanisms, with Rab proteins further assisting in cargo sorting and sEVs release. It is worth noting that sEVs biogenesis mechanism was found to be dysregulated in cancer, leading to a remarkable increase in the number of sEVs released from cancer cell lines (49). In BC, Riches et al. reported that the amount of sEVs released from the BC cell line (B42 clone 16) was evidently higher than that released from the parental normal mammary epithelial cells (HMEC B42) (55). Moreover, the biogenesis of sEVs is influenced by a variety of extrinsic factors in addition to the biological factors mentioned above. These include cell types, cell status, hypoxia, serum conditions, cytokines and growth factors, drugs and radiotherapy (56–59).

## sEVs derived from breast cancer and their role in cancer progression

**Figure 3:**
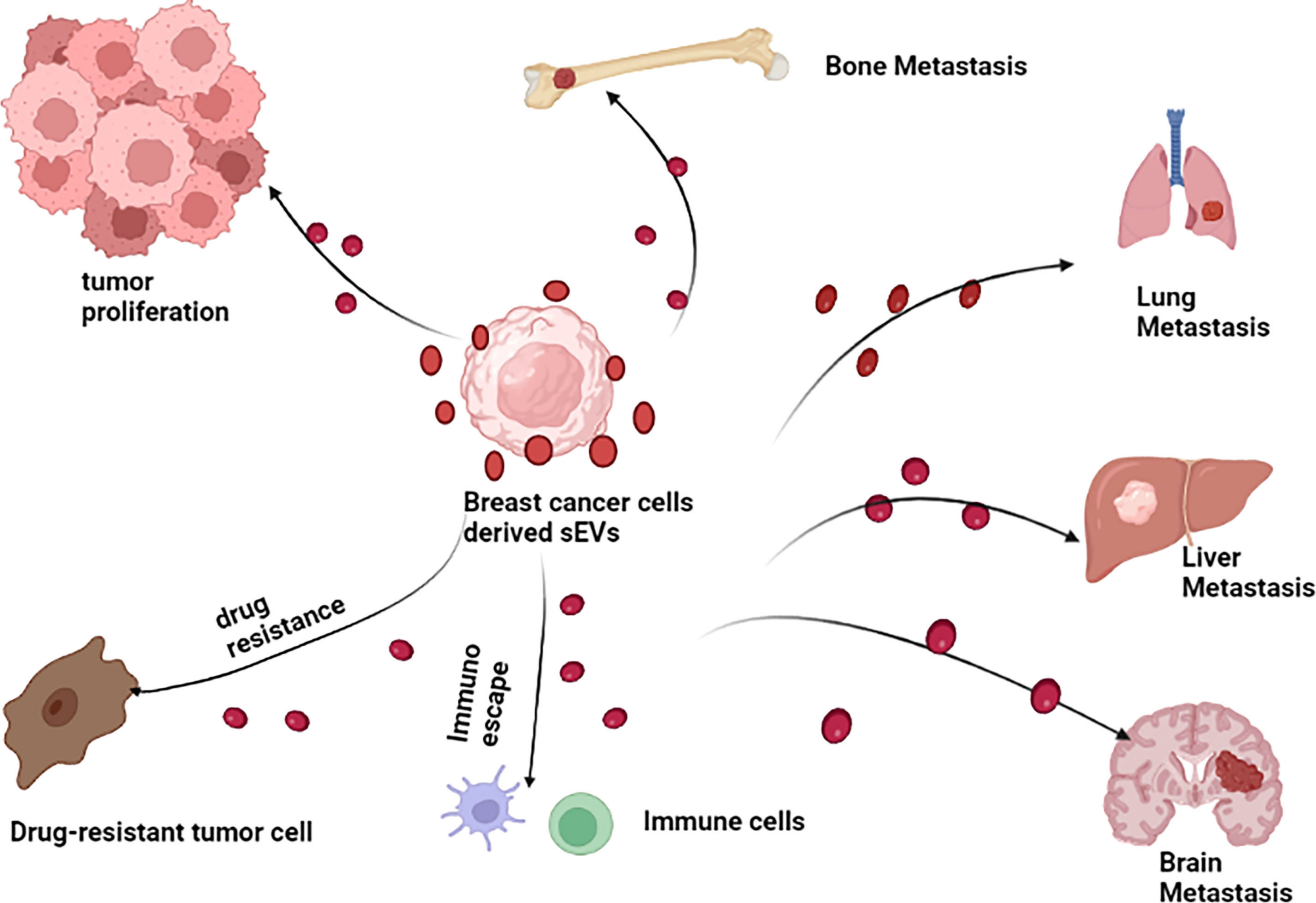
Breast cancer cell derived sEVs have potential role in promoting breast cancer growth, invasion and metastasis, modulating immune escape and drug resistance.

sEVs play a relatively pivotal role in tumorigenesis (60–62). Tumor-derived sEVs are important mediators of intercellular communication between tumor cells and normal stromal cells in local and distant tumor microenvironment (TME), thus promoting tumor development (63). It was verified that sEVs play a multifaceted role in the progress of BC (**Figure 3**). In BC, sEVs can help remodel tumor microenvironment by delivering signaling molecules to cancer cells, in addition to participating in the initial malignant transformation (64). Moreover, several studies have been confirmed sEVs can transfer its functional cargos to target cells, promoting cancer cells proliferation, invasion and metastasis, immune escape and chemoresistance. For example, in an interesting study, Tan et al. showed that sEVs-mediated TGF-β1/Smad pathway further promoted BC cells proliferation and migration by inhibiting apoptosis and enhancing epithelial mesenchymal transition (EMT), which in turn contributes to adriamycin resistance in BC cells (65). Likewise, BC cell-derived cancer-associated fibroblasts (CAF) dramatically promoted BC cells proliferation and migration *via* sEVs (66). Recently, it has been shown that BC cell-derived sEVs contributed to BC bone metastasis. In particular, sEVs facilitated pre-metastatic ecotone production by promoting osteoclast differentiation and enhancing bone metastasis (67). The investigation of sEVs function in BC development and progression is an important topic in order to understand the mechanisms underlying these processes, especially in the search for better and new therapeutic approaches. Therefore, it is imperative to figure out the molecular mechanisms of sEVs in BC. However, as far as we know, those mechanisms have not yet been fully elucidated. Despite the fact that research on BC-related sEVs is still in its early stages, the quantity and quality of studies related to them have been improved in recent years, providing new insights into the mechanisms of BC progression. In the following section, we briefly summarize the crucial role of sEVs in BC proliferation, invasion and metastasis, immune escape, as well as its drug resistance so as to get a comprehensive understanding of the relationship between them.

### Involvement of sEVs in BC proliferation

It has been demonstrated that tumor-derived sEVs contain proteins and nucleic acids cargo molecules that promoted BC cells proliferation. Nischarin is an integrin α5β1-binding protein, the work by Maziveyi et al. found that sEVs enriched with Nischarin-positive cells promoted *in situ* BC cells migration and tumor growth *in vivo* (68). Another recent study in BC was done by Li et al. showed that sEVs-derived miR-1246 is highly expressed in metastatic BC (MDA-MB-231) cells compared to non-metastatic or non-malignant BC cells, and that it promoted BC cells proliferation by targeting cell cycle protein-G2 (CCNG2) (69). Similarly, Zhang et al. showed that long-stranded non-coding RNA transfer-associated lung adenocarcinoma transcript 1 (MALAT1) was highly expressed in BC tissue-derived sEVs and that overexpression of MALAT1 induced BC cells proliferation and tumor size, which was supported by *in vivo* and vitro experiments (70). Additionally, PD-L1 in sEVs suppressed the ability of T cells to kill BC cells and promoted tumor proliferation *in vivo* (71). Recently, *In vitro* findings indicated that sEVs derived from macrophages co-cultured with apoptotic MCF-7 cells increased IL-6 expression in BC cells (MCF-7 cells), which activated the STAT3 signaling pathway and promoted the expression of downstream tumor-proliferating (CyclinD1) genes (72). This suggest that the inhibition of sEVs secretion and the STAT3 signaling pathway activation might be promising approach to block the growth of breast tumors, thus providing new targets for therapeutic treatment. As for what components in the sEVs make them capable of promoting tumor cells proliferation, we need conduct additional research to address the specific mechanisms.

It is notable that pathological factors may influence the role of sEVs in BC proliferation. Due to the rapid growth of the tumor mass, there are hypoxic areas within the tumor where the oxygen concentration is significantly lower than in healthy tissues. Such hypoxic areas are associated with a more aggressive cancer phenotype and worse prognosis (73). Egea et al. found that overexpression of let-7fmiRNA in hypoxic human mesenchymal stem cells (hMSC) promoted the release of sEVs, which resulted in reduced BC tumor proliferation when sEVs were taken up by BC cells (74). Obesity and type 2 diabetes (T2D) are both associated with a higher BC incidence and worse cancer-related outcomes (75, 76). Cao et al. revealed that sEVs from BC directly suppress insulin secretion through miR-122, leading to impaired glycaemic control and enhanced BC cells growth (77). In addition, other studies have shown that sEVs promoted tumor progression through NF-κB pathway (78)、Hedgehog pathway (79) and PI3K-Akt pathway (80). In the same way, subsequent studies have shown that sEVs derived from BC-associated stromal cells can promote proliferation and migration by modulating the Hippo pathway in non-invasive BC cells (81). On the other hand, most of the studies have not deepened in the downstream intracellular signaling triggered by sEVs in BC proliferation, thus leaving the field open for more complete and comprehensive studies.

### Involvement of sEVs in BC invasion and metastasis

The communication between cancer cells and neighboring cells is crucial for tumor development. Metastatic diffusion is the leading cause of BC-related deaths (82). Numerous *in vitro* and preclinical models have demonstrated that the transfer of cargo molecules carried by sEVs between tumor and normal cells effectively contributes to a distal microenvironment conducive to cancer progression (83, 84). In BC patients, the most common sites of metastasis are bone, lung, brain and liver (85). BC brain metastases (BCBM) occurs in approximately 10-30% of patients with metastatic BC, and BCBM is associated with poor prognosis (86). They observed that BC sEVs-derived miR-1290 and miR-1246 activate astrocytes in the brain metastatic microenvironment and that sEVs-derived miR-1290 promotes progression of brain metastases through the novel sEVs-miR-1290→FOXA2→CNTF signaling axis (87). In summary, this indicates astrocytes activated by miR-1290 have the ability to promote progression of BC brain metastases. In addition, this shed new light on the functionality of sEVs and mechanisms by which brain-metastatic BC primes astrocytes in the brain to facilitate BC brain metastasis.

To identify sEVs-bound proteins related to metastasis, Zhang et al. used mass spectrometry to profile sEVs from highly and poorly metastatic BC lines of human and mouse origins. They demonstrated that integrin αv was detected more frequently in circulating sEVs of patients with late-stage (III and IV) than early-stage (I and II) BC. Further analysis revealed integrin αvβ1 is linked to sEVs from metastatic BC cells, whose export into sEVs is in part regulated byβ-Galactose lectin-3(Gal-3) (88). This study highlights the potential of sEVs-bound integrin αvβ1 as a novel prognostic and therapeutic target in BC metastasis. A previous study showed that sEVs enriched with the tetra-transmembrane protein CD81 promoted BC cells metastatic spread (89). Furthermore, Annexin II in primary BC cell-derived sEVs induced angiogenesis *in vitro* and *in vivo via* tissue fibrinogen activator (tPA)-dependent mechanism and promoted lung and brain metastasis (90). Similarly, Shen et al. demonstrated that miR-7641 rich in sEVs play an essential role in BC invasion and metastasis (91). Triple negative breast cancer (TNBC) is a heterogeneous subtype of BC characterized by poor prognosis, high invasiveness and high mortality (92). Li et al. found that sEVs containing CD151 had significantly higher serum expression levels in TNBC patients than in healthy subjects, and they also showed that CD151 dramatically inhibited the invasion and metastasis of TNBC cells (93). Similarly, deubiquitinating enzymes rich in sEVs are highly expressed in TNBC patients and caused BC cells migration and extravasation in a paracrine way (94).All of this evidence suggests that sEVs-derived cargo molecules can mediate tumor progression by promoting BC cells invasion and metastasis. Importantly, further analysis revealed BC-derived sEVs were seen to contribute to metastasis by altering the tissue mechanics of distant organs to support tumor cell invasion and seeding (95). However, the specific mechanisms involved in this process have not yet been elucidated, there is the need for more studies to validate this.

Recently, it was found that sEVs-derived proteins and nucleic acids components enter recipient cells through sEVs uptake pathway and enhanced recipient cells invasion and metastasis by affecting downstream signaling pathways and a series of cascade responses (96). It was shown that CAFs-derived sEVs carrying miR-181d-5p promoted BC cells invasion and migration by targeting cadal-related homeobox 2 (CDX2) and downregulating CDX2 and its downstream gene -homeobox (97). A recently published article claimed that BC-derived sEVs promoted the activation of the Wnt pathway by cancer-associated fibroblasts (CAFs) through the miR-146a/Thioredoxin Interacting Protein (TXNIP) axis, which in turn enhanced BC cells invasion and metastasis (98). Besides, granule protein precursor (PGRN) contributed to BC cells invasion and metastasis by promoting EMT and activating ERK1/2 pathway (99). Kong et al. performed a very interesting experiment in which they found that miR-130a-3p rich in sEVs were abnormally downregulated in human BC tissues and circulating blood, and that low levels of miR-130a-3p expression of sEVs origin were associated with lymph node metastasis and advanced TNM staging. *In vitro*, sEVs-derived miR-130a-3p inhibited human breast cancer stem cells (BCSCs) invasion and metastasis by directly regulating the RAB5B/epidermal growth factor receptor signaling pathway (100). Another study showed that sEVs-derived miR-146a promoted the conversion of normal fibroblasts (NFs) to CAFs through the miR-146a/thioredoxininteracting protein (TXNIP) axis, accelerating BC cells invasion and metastasis (98). The evidence presented in this section suggests that sEVs obtained from BC cells can promote cell invasion and metastasis and are involved in supporting tumorigenesis.

### Involvement of sEVs in BC immune escape

Recent evidence suggests that sEVs also play an important role in remodeling the immune microenvironment of tumors. Since sEVs contain various biomolecules both on their surface and within their own lumen, they can modulate the immune response of immune cells (101). As described earlier, programmed cell death receptor ligand 1 (PD-L1) has been reported to be packaged into the sEVs of tumor cells, and PD-L1 in sEVs enables cancer cells to evade anti-tumor immunity by inhibiting T cell activation (102). Further analysis revealed that miR-92 rich in sEVs derived from breast cancer fibroblasts (CAF) were taken up by cancer cells. MiR-92 targeted Yes-associated protein 1 (YAP1) and their interaction further increased PD-L1 levels in cancer cells, and PD-L1 significantly induced apoptosis and impaired proliferation of T cells, and also prevented the cell killing function of NK cells (103). Another study showed that BC cell-derived sEVs suppressed T cells proliferation *via* transforming growth factor-β (TGF-β) (104). These studies all confirmed that sEVs mediate BC progression through modulation of immune function and also provide clues for further research directions to improve early diagnosis and treatment of BC.

It is worth noting that the mechanisms by which cancer cells escape the immune system mainly include reduced immunogenicity and activation of immunosuppressive signals (105, 106). Myeloid-derived suppressor cells (MDSCs) are involved in tumor growth, in part by suppressing T cells activation through the toll-like receptor (TLR) junction protein MyD88 (107). Existing studies have found that injection of BC cell (TS/A)-derived sEVs into mouse models induced the release of MDSCs from primary tumors and spleens (108). Interestingly, breast tumor-derived sEVs were also found to reduce the immune response by inhibiting NK cells toxicity. In another study, TS/A tumor-derived sEVs were injected into mice and their cytotoxicity of NK cells was reduced by decreasing the percentage and activity of NK cells (109). Recently, Xing et al. demonstrated that deletion of lncRNA X inactive specific transcript (XIST) in BC cells induced the release of miR-503 rich in sEVs from BC cells and that miR-503 promoted microglia transition from M1 to M2 *via* STAT3 and NF-κB pathways, leading to local immune suppression (110). Consistently, Chow et al. found that circulatory sEVs produced by BC cells activated macrophages through NF-kB signaling as well as induced proinflammatory activity by over-producing of different inflammatory cytokines (111). By targeting specific genes associated with immunosuppression in BC-derived sEVs and blocking or reversing the biological functions of both, some therapeutic effects on BC are expected. And emerging evidence indicated that metastatic breast tumor cells release abundant TβRII-positive sEVs and stimulated transforming growth factor-β (TGF-β)/SMAD activation in adjacent pre-malignant tumor cells and remote recipient such as CD8+ T cells. Strikingly, sEVs-TβRII as cargo delivered to CD8+ T cells induced the activation of SMAD3 which cooperates with TCF1 transcription factors to impose CD8+ T cell exhaustion and dysregulation of anti-tumor immunity (112). Together, their findings not only identify a possible mechanism by which BC cells -derived sEVs can promote T cell depletion and suppress host anti-tumor immunity but may also identify immunotherapeutic targets against the most damaging breast tumors. Taken together, these studies suggest that tumor cell-derived sEVs are not merely involved in inducing immune responses, but also suppressing cellular immune responses and converting immune cells into a tumor-supporting phenotype.

### Involvement of sEVs in BC drug resistance

Currently, the most common cause of BC mortality is tumor recurrence due to multidrug resistance (MDR), leading to BC being one of the leading fatal cancers in women (113). Tamoxifen is the most commonly used drug for ER-positive BC (114). However, most BC patients eventually develop tamoxifen resistance and exhibit poor prognosis (115), it poses a considerable therapeutic challenge for BC patients. Recently, miR-9-5p-enriched in sEVs were reported to enhanced the resistance of BC cells to tamoxifen by downregulating its target gene ADIPOQ (116). Moreover, miR-101 rich in sEVs inhibited the phosphatase PTEN and activated Akt by targeting membrane-associated guanylate kinase (MAGI-2), resulting in tamoxifen resistance to BC cells (117). With the development of sEVs research in recent years, sEVs may hold great promise for overcoming multidrug resistance in cancer.

It has been reported that the upregulation of miR-21 rich in sEVs has now been validated *in vitro* and *in vivo* to be closely associated with trastuzumab resistance (118, 119). Trastuzumab is an antibody that binds HER2. Besides conferring resistance, sEVs also reduce the effectiveness of trastuzumab. Ciravolo et al. found that sEVs from serum of HER2-positive BC patients combined with trastuzumab reduced drug effectiveness and suppressed BC cells proliferation (120). Consistent with these results, BC cells (BT-474 and SKBR3)-derived sEVs reduced the trastuzumab-induced toxicity of peripheral blood mononuclear cells (PBMCs) to BT-474 cells (121). sEVs derived from cisplatin-resistant MDA-MB-231 cells are characterized by high expression of miR-423-5p. They transferred cisplatin-resistant phenotypes to recipient cells by promoting the proliferation, metastasis, and anti-apoptotic signaling (122). In addition, sEVs miR-1246 was found to promote epirubicin and gemcitabine resistance by inhibiting Cyclin-G2 in BC cells (69). Consequently, sEVs cargo molecules play a predominant role in reversing BC drug resistance.

Remarkably, it has been shown that the role of sEVs in BC drug resistance can be investigated through the downstream signaling pathways of sEVs cargo molecules and the regulation of target gene expression. It has recently been described that anticancer drug strongly increased tumor cell secretion of sEVs, facilitating the chemoresistance and posttherapy relapse through signaling pathway activation and inflammation induction (123). For example, sEVs containing small nucleolar RNA host gene 14 (lncRNA-SNHG14) promoted trastuzumab resistance in patients with HER2-positive BC. The signal transduction reporter array indicated that the trastuzumab resistance mediated by lncRNA-SNHG14 occurs through the Bcl-2/BAX axis (124). Similarly, sEVs containing lncRNA AGAP2 antisense RNA 1 (AGAP2-AS1) enhanced trastuzumab resistance in BC cells (125). Another study showed that sEVs containing higher expression of miR-770 dramatically inhibited adriamycin resistance in TNBC cells *via* the oncoprotein SNMN1 (126). Importantly, Yang et al. demonstrated that BC patients receiving chemotherapy will activate the EZH2/STAT3 pathway in BC cells, which then secrete miR-378a-3p and miR-378d rich in sEVs, and chemotherapy-surviving BC cells will prompting activation of the WNT/β-catenin and Notch stem cell pathways and subsequently leading to drug resistance (127). BTBD7 is a highly conserved protein, sEVs carrying miR-887-3p could target BTBD7 and activate the Notch1/Hes1 signaling pathway, thereby promoting BC cells drug resistance (128). This study may provide a new understanding of BC treatment in the aspect of cell sensitivity. However, whether other downstream signaling pathways are involved in BC cells drug resistance mediated by sEVs remains unclear.

Recently, Yang et al. analyzed the expression of glutathione S-transferase P1 (GSTP1) in BC cells and tissue-derived sEVs, and they found that GSTP1 enriched in sEVs had the ability to transfer drug resistance. Importantly, GSTP1 was highly expressed in adriamycin-resistant cells and their corresponding sEVs, thus helping to predict clinical chemoresistance (129). As well, sEVs may be associated with resistance to doxorubicin (130) and paclitaxel (131). These data suggest that sEVs play a significant role in drug resistance. Nevertheless, specific mechanisms by which sEVs play a vital role in BC drug resistance process need to be further explored. These studies may contribute to understanding sEVs-mediated delivery of chemoresistance and overcoming chemoresistance in BC therapy. In addition, differentially expressed non-coding RNAs and proteins in sEVs from chemoresistant BC cells support their potential use as disease biomarkers to predict chemotherapy response in BC patients.

## Clinical applications of sEVs in BC

### sEVs as potential biomarkers in BC diagnosis and prognosis

Effective screening tools are essential for early diagnosis and monitoring the prognosis of BC. For this purpose, several biomarkers have been developed, including tissue biomarkers (e.g. hormone receptors, HER2, Ki67), genetic biomarkers(e.g. BRCA1/2) and serum biomarkers (e.g. CA 15.3, CA549) (132). However, these methods are either invasive tumor biopsies or do not provide comprehensive information about the status of the cancer (133, 134), which makes “precision medicine” difficult to achieve. sEVs play a crucial role in intracellular communication by directly binding with surface receptors or transferring their contents to another cell (135). In particular, the lipid bilayer structure of sEVs protects their cargoes from degradation, and the easy availability, stability *in vitro* and real-time assessment of sEVs make them as ideal potential biomarkers (136, 137). Currently, some studies have reported that sEVs cargo molecules can be considered as candidates for early diagnosis and prognosis of BC. For example, Wang et al. artificially investigated whether sEVs could be used as early diagnostic and prognostic indicators for BC, and found significantly higher levels of cargo molecules of sEVs in BC patients than in healthy controls and patients with benign breast tumors using meta-analysis. Moreover, the authors showed that some sEVs proteins (HER2, KDR, CD49d, CXCR4 and CD44) and miRNAs (miR-340-5p, miR-17-5p, miR-130a-3p, miR-93-5p) are associated with tumor recurrence or distant organ metastasis (138).

A previous study has investigated the expression of sEVs enriched in membrane linked Annexin A2 (AnxA2) was substantially higher in the serum of BC patients compared to non-cancerous women. This study also demonstrated that higher levels of expression of AnxA2 rich in sEVs in BC were remarkably associated with tumor stages and poor patients’ survival. Therefore, AnxA2 may serve as a potential diagnostic and prognostic biomarker for BC (139). According to reports, the levels of cancer-associated fibronectin and developmental endothelial locus-1 (Del-1) proteins detected in circulating sEVs were significantly elevated at all stages of BC and returned to normal after tumor removal (140). Another study showed that TNBC-released CSF-1-bearing sEVs promote tumor immune microenvironment associated with a better prognosis in TNBC patients (141).

Apart from proteins, a large number of miRNAs involved in BC progression have been identified in BC-derived sEVs that can be used as biomarkers for them (**Table 1**). MiRNAs in sEVs have been shown to be associated with breast tumor subtypes and staging. MiR-939 rich in sEVs are highly expressed in basal-like tumor subtypes and are associated with poorer prognosis in TNBC (155). Recently, Zou et al. studied the expression of 12 miRNAs in 32 pairs of serum-derived sEVs samples from BC patients and healthy controls. 10 miRNAs (let-7b-5p, miR-106a-5p, miR-19a-3p, miR-19b -3p, miR-25-3p, miR-425-5p, miR-451a, miR-92a-3p, miR-93-5p, and miR-16-5p) were consistently upregulated in serum-derived sEVs from BC patients compared to healthy controls (156). Interestingly, Li et al. demonstrated that miR-148a levels were significantly downregulated in serum sEVs of BC patients compared to healthy patients with benign breast tumors. Additionally, downregulation of miR-148a in serum sEVs was strongly associated with diagnostic staging and disease recurrence, suggesting that it may be a potential non-invasive diagnostic and prognostic biomarker for BC (157). Another study proved that lncRNA CASC9 was significantly upregulated in BC tissues and cells, and it regulates checkpoint kinase 1 (CHK1) by competitively binding to miR-195/497 cluster, which in turn accelerates BC cells proliferation (158). This indicated lncRNA CASC9 may be used as a potential diagnostic biomarker for BC.

**Table 1:** Clinical applications of breast cancer derived sEVs associated MiRs.

Clinical applications	EV contents	EV origin	Tendency	Mechanism of action	References
**Diagnostic biomarkers**	miR-17-5p	Serum	Downregulation	Predicted target genes	(142)
	miR-21-5p	Plasm	Upregulation	Tumor suppressor effect	(143)
	miR-1910-3p miRNA-21-5p and miRNA-10b-5p	Serum Serum	Upregulation Upregulation	promotes proliferation, metastasis, and autophagy of BC cells promotes invasive of BC cells	(144) (145)
	miR-423, miR-424, let7-i and miR-660	Urine	Downregulation	Tumor suppressor effect	(30)
	miR-3613-3p	BC tissues	Upregulation	inhibit BC cells proliferation, ROS production and metastasis by targeting SOCS2.	(146)
	miR-1246	BC cells	Upregulation	miR-1246 could promote invasion in normal HMLE cells partially targeting CCNG2 by binding to its 3’-UTR.	(69)
	miR-424 miR-423 miR-660 let7-i miR-373 miR-101	Serum, Urine, BC tissues	Upregulation	Unknown	(147)
	miR-106a-3p miR-106a-5p miR-20b-5p miR-92a-2-5p	Plasma, Serum, BC tissues	Upregulation	Unknown	(148)
	miR-130a-3p	Blood samples,BC tissues	Downregulation	Directly regulate RAB5B/EGFR signaling pathways	(100)
**Therapeutic target**	miR-9 miR-181a	BC cells, BC tissues	Upregulation	miR-9 and miR-181a activated the JAK/STAT signaling pathway *via* targeting SOCS3 and PIAS3 respectively, resulted in T-cell immunity inhibition and tumor progress.	(149)
	miR-7641	Plasma	Upregulation	Promotes BC progression and metastasis	(91)
	miR-27a-3p	BC cells, BC tissues	Upregulation	Promoted immune evasion of BC cells by activating the PTEN/AKT/PI3K axis.	(150)
	miR-20a-5p	BC tissues, BC cells	Upregulation	Promoted osteoclast formation and bone metastasis by targeting SRCIN.	(151)
	miR-22-3p	BC cells	Upregulation	Mediate tumor vessel abnormalization by suppressing transgelin	(152)
	miR-567	Serum, BC tissues	Deregulation	Reversing trastuzumab resistance *via* regulating autophagy	(153)
	miR-146a	BC cells	Upregulation	Enhance the transformation of NFs into CAFs *via* TXNIP/Wnt pathways	(98)
	miR-455-5p miR-1255a	BC cells	Upregulation	miR-455-5p may exert tumor promoting roles by inhibiting the expression of CDKN1B and influencing cell cycle and miR-1255a may be oncogenic by down-regulating SMAD4 and affecting TGF-β signaling pathway, which resulted in poor prognosis.	(154)

In particular, sEVs-encapsulated miRNAs can be used as prognostic biomarkers for BC metastasis progression. The work by Curtaz et al. identified hsa-miR-576-3p was significantly upregulated, and hsa-miR-130a-3p was significantly downregulated in sEVs from BC patients with cerebral metastases. This suggest that the two miRs with the potential to serve as prognostic biomarkers for brain metastasis in BC (159).On the other hand, sEVs-derived miRNAs are of great significance in predicting TNM staging, worsening and poorer prognosis of BC types. For example, miR-197, miR-29b-2, miR-205 and miR-155 rich in sEVs were associated with lymph node and tumor size correlated with lesion metastasis, and the presence of miR-205 and miR-155 rich in sEVs suggested distant metastasis of lesions (160). Consequently, controlling the expression of cargo molecules in sEVs may be a feasible approach to protect BC high-risk individuals.

In ongoing clinical trials, sEVs are being evaluated as biomarkers for early diagnosis and prognosis. For example, a clinical trial is exploring the uses of sEVs-HSP70 for the diagnosis of early BC (161). Research on sEVs as molecular biomarkers is ongoing, and their biological properties are facilitating clinical applications.

In conclusion, these studies confirm the great potential of sEVs as diagnostic biomarkers for BC, as they are highly representative of the inclusion’s characteristic of the cells from which they originate. However, no specific cargo of sEVs is currently available for clinical applications. Accordingly, more accurate and robust studies of sEVs are needed. It is worth noting that additional functional analysis and careful validation of the identified biomarkers is warranted prior to the application of sEVs for diagnosis.

### sEVs as potential therapeutic targets

BC treatment is multidisciplinary, the main treatment modalities are surgery, chemotherapy, radiotherapy, endocrine therapy, immunotherapy, and targeted therapy, depending on cancer subtypes and stages (162–164). Notably, chemotherapy with targeted agents has been widely accepted as a standard of care, especially in HER2-positive BC and TNBC. Moreover, patients in the premenopause with hormone receptor-positive, HER2-negative, lymph node-positive breast cancer benefit from combined endocrine therapy and chemotherapy (165). The most common treatment among BC patients at an early stage is breast conserving surgery with adjuvant radiation therapy, while women diagnosed with metastatic disease often receive radiation and/or chemotherapy (166). It is worth noting that these methods have a high risk of recurrence and side effects (167). Moreover, high resistance to chemo- or radiotherapy weakens the therapeutic efficacy (168).Recently, neoadjuvant chemotherapy (NACT) has been reported to be one of the most common treatments for BC and has been shown to be effective in treating patients with locally advanced BC, helping to improve their quality of life. However, there are still some patients who are not sensitive to neoadjuvant chemotherapy regimens, leading to delayed disease, missed optimal treatment or overtreatment (169). On the other hand, there are a considerable number of studies demonstrating circulating tumor cells (CTCs) detection as an effective technique for the evaluating BC treatment efficacy and recurrence (170). Jacot et al. revealed that unlike PD-L1(+) tumors, PD-L1 expression in CTCs was associated with survival in metastatic BC, indicating a potential role of PD-L1(+)-CTCs as a stratifying factor for anti-PD-1/PD-L1 treatment for metastatic BC patients (171). However, due to the number of CTCs in blood is low and profoundly diluted by blood cells, making their detection technically difficult particularly in early BC (172, 173). Consequently, there is an urgent need to identify more effective molecular targets to improve BC treatment.

As natural carriers, sEVs protect cargoes from degradation or neutralization *in vivo*, and sEVs have high biocompatibility, low toxicity and low immunogenicity (174, 175). With appropriate modifications, the stability and efficiency of treatment can be improved and can enhance the uptake of target cells (176). Of significance, engineered sEVs that can be used as therapeutic agents to decelerate disease progression are becoming a hot topic of research.

Furthermore, BC derived sEVs associated MiRs can also be used as therapeutic targets (**Table 1**). A previous study showed that sEVs isolated from bone marrow mesenchymal stem cells successfully delivered anti-miR-142-3p in BC tumors *in vivo*, restoring the expression levels of their target genes APC and P2X7R, thereby reducing the tumorigenicity of BC *in vitro* and *in vivo* (22). MiR-3182 rich in sEVs derived from human umbilical cord mesenchymal stem cells (HUCMSCs) suppressed the invasive process of TNBC *in vitro*, suggesting that miR-3182 may be used as a therapeutic modality for TNBC (177). Another recent study demonstrated lncRNA DARS-AS1 overexpression dramatically enhanced the migration and invasion of TNBC tumors by inhibiting miR-129-2-3p to activate the NF-κB/STAT3 signaling pathway both *in vitro* and *in vivo*. Treatment with DARS-AS1 siRNA-loaded exosomes (EXOs) remarkably slowed TNBC cells growth and liver metastasis. This study suggested that siRNA-loaded EXOs may be used as native nanocarriers to deliver siRNAs for TNBC therapy (178). In particular, Senigagliesi et al. showed that TNBC-derived sEVs are able to directly modify MCF7 cells by inducing a decrease in cell stiffness, rearrangements in cytoskeleton, focal adhesions and nuclear/cellular morphology, and an increase in Yap downstream gene expression (179). This revealed that testing the biomechanical response of cells after sEVs addition might represent a new functional assay that can be exploited for future applications both in BC diagnosis and therapy.

To assess the effect of sEVs derived from TNBC cells on the cytotoxicity of therapeutic agents in non-tumorigenic breast cells (MCF10A), treatment of MCF10A cells with sEVs derived from HCC1806 cells, a TNBC cell line, significantly increased MCF10A cells proliferation and induced resistance to paclitaxel and doxorubicin in MCF10A cells by Ozawa et al. (180)

Recently, it has been reported that loading of miRNA-containing sEVs is a possible way to inhibit BC cells invasion and metastasis. For example, by using tumor-derived sEVs as carriers, Moradi-Chaleshtori et al. transported miR-130-enriched in sEVs to M2 macrophages, which in turn reduced BC cells proliferation, migration, and invasion (181). In addition, BC stem cells-derived extracellular vesicles (BCSCs-EVs) facilitated epithelial-mesenchymal transition (EMT) of BC cells by delivering miR-197 to BC cells and inhibiting PP ARG expression, thereby promoting growth and metastasis of BC cells (182). Interestingly, the chemotherapy-based immunotherapy is emerging as a promising therapeutic approach. Zhao et al. first reported docetaxel (DTX) as chemotherapeutic modularity was loaded into M1 macrophage-derived sEVs(M1- sEVs) with M1 proinflammatory nature to establish DTX-M1-sEVs drug delivery system. They revealed that DTX-M1-Exo promoted polarization of naïve macrophages to M1 phenotype and maintained M1 form upon M2 stimulation through modulating mitochondrial function. Importantly, DTX-M1-sEVs has been demonstrated significantly improved the anti-cancer therapeutic efficacy with minimal side effect (183). Therefore, these trials showed encouraging results that sEVs may serve as early predictors for therapeutic response.

Actively loading cargo into donor cells is a major component of sEVs engineering (184). Kim et al. found that erythroleukemic K562 cells containing human leukocyte antigen (HLA)-A2, CD80, CD83, and 41BBL co-stimulatory molecules were engineered to secrete sEVs that activated CD8+ T cells to enhance the effectiveness of immunotherapy (185). In addition, new engineered EVs platforms such as synthetic EVs-like synthetic nanoparticles and EVs-mimicking nanocapsules are gradually being developed (186, 187). This all indicates that sEVs are a promising and rapidly developing area of research in disease treatment. However, a further exploration of the full potential of sEVs in BC therapy is needed to make them more effective and more widely available for use in clinic. There is also urgent need to establish reliable assays to assess the therapeutic potential of sEVs and to develop these assays into formal efficacy tests for clinical applications (188). These key studies demonstrated that sEVs is promising to direct therapy. Importantly, this also confirmed sEVs role in dynamic and real-time monitoring of BC treatment. However, there is the need for more studies to validate this.

## Conclusion

In recent years, sEVs have received much attention due to their pathophysiological role in tumor progression. However, the understanding of the mechanism and clinical applications of sEVs in BC are still lacking. Tumor invasion and metastasis, immune evasion, and drug resistance are the main obstacles in treating advanced BC. Since sEVs can act as a bridge for cellular communication in the tumor microenvironment, they lead to tumor development, invasion, metastasis and drug resistance. Elucidating sEVs participation in the various steps of BC development, encompassing initiation, progression, immune escape, resistance to treatment, and recurrence after a period of remission is important. Therefore, the study of sEVs participation in BC is expected to provide a platform and a guide for developing novel diagnostic and prognostication tools and efficacious treatments. Further studies of sEVs will contribute to a more comprehensive and multidimensional understanding of BC. This study expands our understanding of the function and tumorigenesis mechanisms of sEVs and provides a new perspective for the diagnosis and prevention of BC. The mechanism of BC-associated sEVs depends mainly on their complex cargo molecules, which can fuel cancer cells, contribute to their proliferation, invasion and metastasis, induce metabolic reprogramming of cancer cells, and thus mediate the process of BC development and progression, indicating their value as BC biomarkers. As a biomarker, sEVs can provide abundant, stable, sensitive and specific biological information, and are a liquid biopsy specimen with higher application value (189). In addition, sEVs are emerging as valuable therapeutic targets closely related to the development of precision medicine. sEVs can be used as drug carriers with good biocompatibility, which can be easily absorbed by target cells and lead to effective therapeutic effects (190).

Although considerable progress has been made in understanding sEVs and their cargoes, a number of challenges are still present. First, there is still no gold standard method for the isolation and identification of sEVs, no ideal high purity and efficient strategies for sEVs isolation have been developed yet, resulting in less reproducible or persuasive results. Second, how and when do sEVs reach pre-metastatic ecological sites during disease progression? How do cancer cells acquire the ability to release specific sEVs cargoes that maintain BC plasticity and tumor metastatic spread? Determining which sEVs sources are safe and biocompatible for drug delivery systems in therapy remains an ambiguous issue, the drug delivery modalities and targeted modification techniques of sEVs need further refinement in clinical applications. In addition, the assessment and characterization of circulating sEVs in BC patients with different disease stages should be addressed. Finally, we must further explore the mechanisms of sEVs biogenesis and sorting to effectively design sEVs with specific nucleic acids, proteins or even exogenous drugs. In conclusion, sEVs represent an attractive area of research that remains to explore new opportunities in BC prevention, diagnosis and therapeutic approaches, but there are still some obstacles to overcome before sEVs are ready for clinical use.

## Author contributions

XY searched for literature and wrote the manuscript. DH, TY, ZL, XW, MZ, and JW were involved in edited the manuscript. TZ supervised the project and contributed to the revision of the final manuscript. All authors contributed to the article and approved the submitted version.

## Funding

This work was supported by the Key R&D Planning Project of Jiangxi Science and Technology Commission, China (No. 20203BBGL73126). We thank Yaojiang Que edited the figure.

## Conflict of interest

The authors declare that the research was conducted in the absence of any commercial or financial relationships that could be construed as a potential conflict of interest.

## Publisher’s note

All claims expressed in this article are solely those of the authors and do not necessarily represent those of their affiliated organizations, or those of the publisher, the editors and the reviewers. Any product that may be evaluated in this article, or claim that may be made by its manufacturer, is not guaranteed or endorsed by the publisher.
